# Partial oxidation of methane over SSZ-39 zeolites containing iron, copper, and iron–copper mixtures with hydrogen peroxide: selective control of oxygenate formation

**DOI:** 10.1039/d5ra04892c

**Published:** 2025-11-25

**Authors:** Jeewan Pokhrel, Daniel F. Shantz

**Affiliations:** a Department of Chemical and Biomolecular Engineering, Tulane University 6823 St. Charles Avenue New Orleans LA 70118 USA dshantz@tulane.edu

## Abstract

Here iron and copper containing zeolites are reported for the liquid phase oxidation of methane to methanol using hydrogen peroxide (H_2_O_2_) as an oxidant. Iron-exchanged SSZ-39 favors formic acid formation while Fe, Cu-SSZ-39 samples with low iron contents shift the selectivity towards methanol with no observable formic acid formation. It was also observed that how the metals are loaded into the zeolite is integral to their catalytic performance. A two-step method wherein iron-exchanged SSZ-39 had copper added to it (Cu/Al = 0.196 and Fe/Al = 0.07) showed promising results compared to other approaches of loading both metals, resulting in a methanol production rate of 5.4 mmol (g_cat_^−1^ h^−1^) after one hour of reaction time. Interestingly, varying the Fe/Cu ratio of the samples enabled the possibility to increase the amount of oxygenates and shift the selectivity. The most active catalyst was Fe, Cu-SSZ-39(t) with Fe/Al = 0.212, Cu/Al = 0.031 that produced formic acid and methanol at a rate of 11.2 and 15.3 mmol (g_cat_^−1^ h^−1^). In general, it is observed that Fe-SSZ-39 and Fe, Cu-SSZ-39 produce more oxygenates than Fe-ZSM-5 and Fe, Cu-ZSM-5 under the same experimental conditions. Analogous to the gas phase oxidation of methane to methanol, the steric constraints of the small-pore zeolite could be one possible reason for this.

## Abstract 2

Here iron and copper containing zeolites are reported for the liquid phase oxidation of methane to methanol using hydrogen peroxide (H_2_O_2_) as an oxidant.

## Graphical abstract

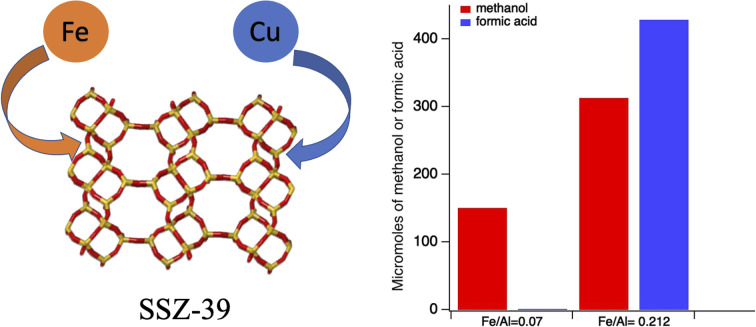

## 1 Introduction

The abundance of methane due to the discovery of shale gas, coupled with the necessity to flare a large amount of gas, increased global warming, and challenges of liquefaction and on-site conversion for its transportation has led to the exploration of new chemical routes for methane utilization.^1,2^ Methane upgrading industrially currently involves steam reforming, an energy intensive process (Δ*H*°_rxn_ ∼205 kJ mol^−1^) to produce syngas which is subsequently converted to hydrocarbons or to methanol and then other chemical compounds, *i.e.*, it is an indirect process.^3^ Alternatively, the direct conversion of methane to methanol holds intrigue as it is exothermic and potentially much less energy and capital-intensive. As a result, it has been intensely studied for many years.^4,5^ Methanol is an energy-dense liquid that can be easily transported and can be used in the production of dimethyl ether,^6^ formaldehyde,^7^ and light olefins^8,9^ or gasoline^10^ through the MTO/MTG process. However, the structural chemistry of methane poses challenges for selective oxidation of methane to methanol. This is due to its low polarity, high C–H bond strength (∼440 kJ mol^−1^) and resulting large activation energy for cleavage of the C–H bond.^5^ Even when the energy barrier is overcome the partial oxidation of methane to methanol is challenging thermodynamically as the C–H bond in methanol is comparatively weaker and complete oxidation to carbon dioxide is thermodynamically favored. Thus, there is a need for the development of a catalyst that will cleave the primary C–H bond and simultaneously selectively oxidize methane to methanol.

The literature on the range of materials that have been investigated for this chemistry is voluminous. Our interest is in transition metal-exchanged zeolites, especially iron- and copper-exchanged zeolites. These systems have been studied by the zeolite community and found to generate a similar ligand field environment within the zeolite cavities that can replicate the activity of the active metal centers of methane monooxygenase by producing metal–oxygen species to attack the strong C–H bonds of methane.^11–13^ However, these sites were believed to only operate stoichiometrically until Román-Leshkov and co-workers demonstrated the existence of sites operating catalytically at steady state but with low conversion (<0.1%) for partial methane oxidation where only O_2_ was used as oxidant over different copper-containing zeolites.^14^ Theoretical studies predict that above 0.2% conversion, methanol undergoes overoxidation at isolated Cu^2+^ sites generating CO_2_ and H_2_O.^15^ The protection of methanol by the formation of more stable products resistant to subsequent oxidation and enabling 100% selectivity to methanol formation has been explored for a long time but still high methanol yields and inhibiting excess oxidation remain as challenges in methane to methanol oxidation using zeolite-based catalysts.^16^

Copper-containing zeolites with small pores such as SSZ-13, SSZ-16, SSZ-39, and SAPO-34 were reported to exhibit better methane reactivity and methanol selectivity than Cu-ZSM-5 and Cu-MOR.^16^ These small pore zeolites are crystallographically much simpler, have lower framework densities (15.1 T-atoms nm^−3^) than MFI, and higher micropore volumes (0.28 cm^3^ g^−1^) that can potentially be twice as large as the micropore volume of ZSM-5. Cu-ZSM-5 had the highest specific activity and STY among medium- and large-pore zeolites, while small-pored zeolites SSZ-13 and SAPO-34 showed higher STY than Cu-ZSM-5 at 210 °C.^17^ Pappas and co-workers studied methane oxidation to methanol over Cu-SSZ-13 (CHA) and obtained methanol productivity of 0.2 mol_CH_3_OH_·(mol_Cu_)^−1^.^18^

The use of iron-exchanged zeolite catalysts in a cyclic process to produce methanol was demonstrated by Panov and co-workers with N_2_O as oxidant. The three-step cyclic process was initiated by the activation of the iron active site with N_2_O at high temperatures, which results in the generation of a high valent iron species. This is followed by the activation of methane on the active sites to form CH_3_O- and –OH species at lower temperatures and finally the extraction of methanol with water. However, this process was quasi-catalytic below 473 K, but continuous methanol desorption occurred above 473 K.^19^

Another approach using iron-containing zeolites employing H_2_O_2_ as the oxidant at low temperatures (∼323 K) was reported by Hammond and coworkers.^20–23^ The catalytic cycle begins with the formation of methyl hydroperoxide which decomposes to form methanol and finally formic acid. Formaldehyde (HCHO) and its hydrated form (H_2_C(OH)_2_) were also observed as short-lived intermediates which rapidly oxidized to formic acid (HCOOH) in a similar study of methane oxidation over Fe-ZSM-5 with H_2_O_2_ by Al-Shihri *et al.*^24^ Based on spectroscopic characterization and DFT simulations, extra-framework diiron species [Fe_2_(µ_2_-OH)_2_(OH)_2_(H_2_O)_2_]^2+^ containing antiferromagnetically coupled high-spin octahedral Fe^3+^ centers were proposed as the active species.^21^ The mechanism involves the formation of a diiron site that coordinates with H_2_O_2_ and forms a Fe^4+^/Fe^2+^ dimer upon rearrangement. A second H_2_O_2_ binds to the Fe^2+^ site, leading to a unique, bifunctional oxidation center. The iron oxo group activates C–H bonds producing methyl radicals, which are captured by the hydroperoxyl ligand at the second Fe center. The active site is regenerated by releasing the methyl hydroperoxide into the solution. Kinetic studies showed that H_2_O_2_ is involved in the rate-determining step, with an activation energy of 61 kJ mol^−1^, similar to that of the soluble methane monooxygenase.^20–22^ Addition of Cu^2+^ to the zeolite improved methanol selectivity creating more ˙OH radicals, facilitating CH_3_OH formation while eliminating HCOOH *via* overoxidation, and the active sites are based on mononuclear Fe^4+^=O sites.^20,25^

**Fig. 1:**
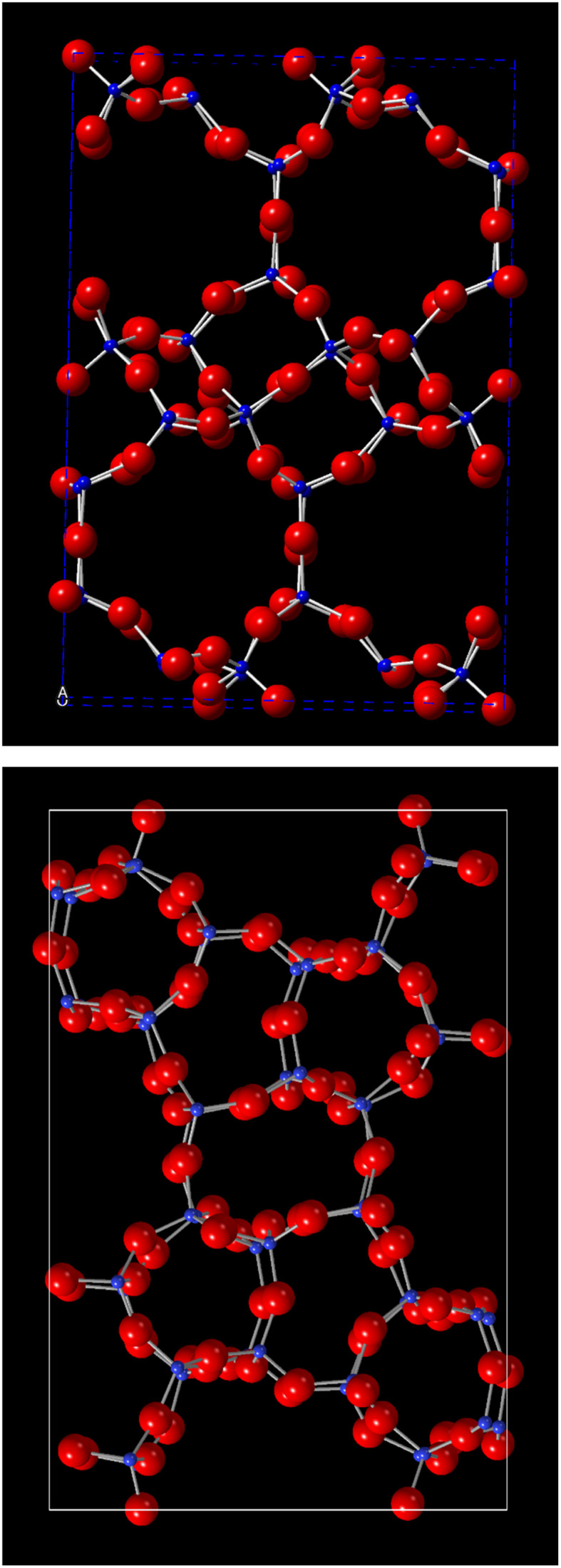
Framework topologies of AEI (top) and MFI (bottom).

Here we report the use of iron- and copper-exchanged SSZ-39 zeolite for liquid phase oxidation of methane to methanol using H_2_O_2_ under high pressure and low temperature conditions. This was in part motivated by the lack of work exploring small-pore zeolites in this process. Also, prior work from our lab has reported on the gas-phase activity for methane to methanol over Cu-SSZ-39.^26^ Subsequent work led to us explore Fe,Cu-SSZ-39 for the same reaction, which proved to be a poor catalyst for the gas-phase oxidation with O_2_. In contrast, this material displayed interesting properties in oxygenates formation productivity and selectivity over SSZ-39 which we describe here and compare to prior work by Hammond and co-workers for Fe,Cu-MFI (Fig. 1).

## 2 Experimental

### 2.1 Materials

Sodium silicate (PQ Brand N, 28.9 wt% SiO_2_, 8.9 wt% Na_2_O), Faujasite (FAU) (SiO_2_ : Al_2_O_3_ = 5.2 : 1, Zeolyst), NH_4_-ZSM-4 (Si/Al = 11.15), and sodium hydroxide pellets (ACS grade) were purchased from VWR. 3,5-dimethyl piperidine (*cis*/*trans* mixture, 97%, Alfa Aesar), methyl iodide (99%, Sigma Aldrich), potassium carbonate (ACS grade, VWR), and methanol were used for the organic structure-directing agent (OSDA) synthesis. Tetrapropylammonium hydroxide (40 wt%) was purchased from Alfa Aesar. Ammonium nitrate (ACS, 95%), copper(ii) sulfate pentahydrate (ACS, 98%) were obtained from Alfa Aesar and iron(ii) sulfate heptahydrate (ACS, 99%), iron(iii) nitrate nonahydrate (98%, metal basis) were obtained from Thermo Scientific. Hydrogen peroxide (3% w/w) and acetonitrile (Hisolv) were obtained from VWR. All chemicals were used as received. Methane (≥99.99%, O_2_ ≤ 5 ppm (v/v)), helium (≥99.999%, O_2_ ≤ 1 ppm (v/v)), hydrogen (100%), nitrogen (≥99.999%), NO (0.9709%, total NO_*x*_ 0.9753%, balance N_2_) and air were provided by Airgas.

### 2.2 Zeolite synthesis

#### 2.2.1 OSDA synthesis

**Scheme 1:**
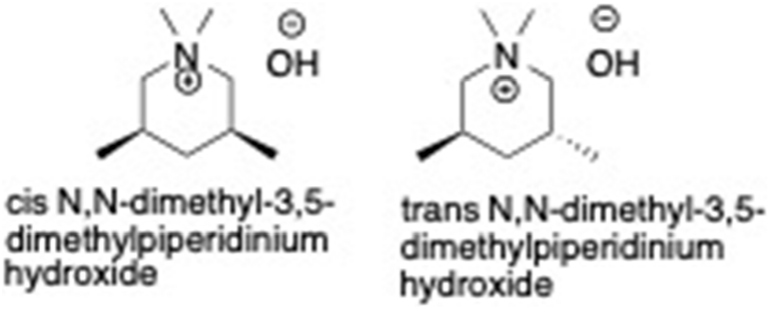
Isomers of the OSDA used in this work.

The OSDA was prepared as adapted from Wagner *et al.*^27^ 28 g (250 mmol) of potassium carbonate was dissolved in 200 mL of methanol and to this mixture, 13 mL (100 mmol) of 3,5-dimethyl piperidine (*cis*/*trans* mixture) was added. Then, 18 mL (300 mmol) of methyl iodide was added gradually under stirring and reacted in the absence of light for 24 h at room temperature. The solution was filtered, and the residual solids were washed with 100 mL methanol. The filtrate was rotavapped to obtain the dry crude OSDA which was recrystallized at reflux using methanol. The recrystallized OSDA (iodide form) was ion-exchanged to the hydroxide form in a column filled with Dowex Marathon A resin. The exchanged OSDA was then titrated with 0.05 M HCl using phenol red as an indicator to determine the concentration of OSDA (0.5–1.0 M). The resulting SDA obtained from this process typically had a molar *cis*/*trans* ratio of 95/5 (Scheme 1).

#### 2.2.2 SSZ-39 synthesis

Zeolite SSZ-39 was prepared per prior literature *via* hydrothermal synthesis with a gel molar composition of 1 SiO_2_ : 0.033 Al_2_O_3_ : 0.57 NaOH : 0.14 R^+^OH^−^ : 28 H_2_O.^28^ As a typical example, 8.66 g of sodium silicate, 1 mL of 1 M NaOH solution, 5 mL (95% *cis*) OSDA solution and finally 0.34 g FAU were added into a Teflon jar.^29^ The resulting mixture was stirred for 2 h at room temperature, and then transferred into two Parr Teflon-lined autoclaves. The synthesis was performed at 140 °C under rotation for 72 h. The samples were then cooled down, filtered, and washed with deionized water (100 mL each) three times until the filtrate had a pH of approximately 7. The samples were then dried overnight at 80 °C. The dried samples were calcined a static muffle furnace at 550 °C in air (heating rate 10 K h^−1^) for 8 h to remove the OSDA.^30^ All calcinations described below were performed in the same way.

#### 2.2.3 One-pot synthesis of Fe-SSZ-39 ^31^

In a separate hydrothermal synthesis, Fe-SSZ-39 was prepared by adding Fe salt together with the precursors in the zeolite synthesis gel. A typical synthesis utilized 152.8 mg Fe(NO_3_)_2_·9H_2_O with gel composition of 1 SiO_2_ : 0.033 Al_2_O_3_ : 0.01Fe : 0.57 NaOH : 0.14 R^+^OH^−^ : 28 H_2_O. All other steps were the same as those used for SSZ-39 and the samples are denoted as Fe-SSZ-39(o).

### 2.3 Ion-exchange

#### 2.3.1 Copper exchange

The calcined zeolites were first exchanged with 0.1 M ammonium nitrate solution (250 mL g^−1^ zeolite) for 8 h at 80 °C. The resulting solids were washed *via* filtration and then dried. This material referred to as NH_4_-SSZ-39. In some cases, this material was then exchanged with 50 mM CuSO_4_ solution (200 mL g^−1^ NH_4_-SSZ-39) for 1 h at room temperature. The mixture was filtered, washed, and dried at 80 °C to yield Cu-SSZ-39.

#### 2.3.2 Iron exchange methods

Multiple approaches were used to load iron into the zeolite and are described below. As mentioned above Fe-SSZ-39 was also obtained *via* a one-pot synthesis wherein iron was included in the synthesis gel. Those samples are denoted as Fe-SSZ-39(o).

##### 2.3.2.1 Gao Method^32^

This method uses NH_4_-SSZ-39 as a starting material where the suspension is prepared by using 1 g NH_4_-SSZ-39 in 100 mL of DI water with the pH maintained at approximately 3.0. The suspension was then heated to 80 °C under bubbling argon to flush oxygen out of the solvent. FeSO_4_·7H_2_O (0.005 mol, 0.139 g) powder was then added into the suspension at 80 °C and exchanged for 1 h. After that time the mixture was cooled down, filtered, washed with deionized water, dried at 110 °C and finally calcined at 550 °C for 8 h to obtain Fe-SSZ-39, which is denoted as Fe-SSZ-39(g).

##### 2.3.2.2 Wet impregnation method^33^

Iron exchanges of SSZ-39 were carried out through a method adapted from Kalamaras *et al.* In a typical experiment, 700 mg of NH_4_-SSZ-39 was taken and 0.0001 M Fe(NO_3_)_3_·9H_2_O (2.1 mL g^−1^ zeolite) was added and exchanged for 6 h at room temperature. After gradual evaporation of water at 70 °C for 4 h, the resulting solids were then dried at 110 °C and calcined at 550 °C to obtain Fe-SSZ-39, which is denoted as Fe-SSZ-39(w).

##### 2.3.2.3 Fe-exchange of commercial ZSM-5

Commercial ZSM-5 in the NH_4_ form (Si/Al = 11.5) was exchanged with 0.05 M Fe(NO_3_)_3_·9H_2_O (100 mL g^−1^ zeolite) at room temperature for 20 h. After the exchange, samples were centrifuged and washed with DI water, dried at 110 °C, and calcined at 550 °C. These samples are denoted Fe-ZSM-5(c).

#### 2.3.3 Mixed iron–copper SSZ-39

Multiple methods were used to generate samples of SSZ-39 containing both iron and copper, detailed below. To aid the reader, the sequence of the elements is the same as the order of the addition of the metal, *i.e.*, first metal, second metal-SSZ-39.

##### 2.3.3.1 Two Step Method^32^

This method uses Fe-SSZ-39 described in 2.3.2.1 (Gao method) as a starting material. Fe-SSZ-39(g) is subsequently copper exchanged with 50 mM CuSO_4_·5H_2_O at room temperature as mentioned in the Cu-exchange section to obtain Fe,Cu-SSZ-39, which is denoted Fe,Cu-SSZ-39(t). This method adds iron *via* the Gao method first then copper *via* the normal protocol.

##### 2.3.3.2 Modified Gao Method^32^

This method uses Cu-SSZ-39, mentioned in 2.3.1. as a starting material. 1 g of Cu-SSZ-39 was dispersed in 100 mL of deionized water and the pH was adjusted to approximately 3.0 by adding 0.1 M HNO_3_. The suspension was then heated to 80 °C under bubbling argon to flush oxygen out of the solvent. FeSO_4_·7H_2_O (0.005 mol, 0.139 g) powder was then added into the suspension and exchanged for 1 h. After that, the mixture was cooled down, filtered, washed with DI water, dried at 110 °C, and finally calcined at 550 °C for 8 h to obtain Cu,Fe-SSZ-39, denoted as Cu,Fe-SSZ-39(m). This sample is made *via* normal copper exchange, then the Gao method is used to add iron.

##### 2.3.3.3 Mixed-metal exchange with Fe and Cu

In this method, copper and iron were simultaneously exchanged into the zeolite. A typical exchange used 1 g NH_4_-SSZ-39 exchanged with 0.05 M Fe(NO_3_)_3_·9H_2_O and 0.05 M Cu(NO_3_)_2_·5H_2_O solutions (basis 100 mL g^−1^ zeolite) for 12 h at room temperature. The products were then filtered, washed, dried at 110 °C, and calcined at 550 °C to obtain Fe,Cu-SSZ-39, denoted as Fe,Cu-SSZ-39(mm). The sample is made by simultaneous exchanges of iron and copper.

### 2.4 Characterization methods

#### 2.4.1 Analytical

X-ray diffraction (XRD) measurements were performed with a Rigaku Benchtop Miniflex 600 X-ray diffractometer with Cu Kα (*λ* = 1.5418 Å) radiation operating at 40 kV and 15 mA. A scanning rate of 0.25° min^−1^ and a step size of 0.02°/step were used over the 2*θ* range of 5–50°. Nitrogen adsorption experiments were performed on a Micromeritics ASAP 2020 instrument at 77 K. Approximately 50 mg of calcined samples were degassed at 573 K for 18 h before analysis. A single-point free space analysis was run at *p*/*p*_o_ = 0.05 to determine cold and warm free space and quantity adsorbed before full micropore analysis. Field-Emission Scanning Electron Microscopy (FE-SEM) images were taken with a Hitachi S4800 high-resolution scanning electron microscope at 3 kV and elemental composition data mainly, Si, Al, Cu, and Fe content were obtained on a Hitachi S3400 electron microscope equipped with an Oxford Instruments detector at 20 kV.

### 2.5 Catalytic testing

High-pressure Parr reactors^34^ equipped with a pressure transducer, thermocouple, and external temperature control were used for the liquid phase oxidation studies with hydrogen peroxide as an oxidant.^20,35^ 27 mg of zeolite catalyst were added to 10 mL of 0.5 M H_2_O_2_ in a Teflon vessel and sealed into a 50 mL batch reactor vessel. Then, the vessel was purged with methane at 25 psi for 5 min to remove any air/oxygen. The vessel was then pressurized with methane to 443 psi and heated to 55 °C. The reaction was carried out for 30 min under stirring at ∼1400 rpm. The vessel was then cooled to ∼20 °C using an ice bath before releasing the gaseous compounds. The solution was filtered, and the liquid products were analyzed by proton NMR.^35^

#### 2.5.1 Quantifying experiments with NMR

For the NMR spectroscopy measurements, the liquid product was added with D_2_O solvent and analyzed using a Bruker DRX instrument operating at 500.13 MHz for ^1^H. The spectra were collected at a frequency of 500 MHz with a relaxation delay of 2 s and receiver gain (RG = 1). To minimize the water peak and quantify the peaks of methanol and other products, solvent suppression was applied with acetonitrile as an internal standard and calibrated against methanol and formic acid. In a typical experiment, 280 µL of sample and 250 µL of D_2_O were loaded in an NMR tube along with the co-insert containing 60 µL of 0.25 vol% acetonitrile. The detected oxygenated species were mainly methanol (*δ* = 3.25 ppm), methyl hydroperoxide (*δ* = 3.75 ppm), and formic acid (*δ* = 8.19 ppm). Note the amount of methanol and formic acid produced below are arrived at by scaling the numbers from the NMR measurement (*i.e.*, micromoles of X/280 µL) to the reaction mixture volume of 10 mL. To obtain the rate of oxygenate production (mmol per gram catalyst per hour) this total amount of oxygenate produced is divided by the product of the time and catalyst mass. The total amount of oxygenate produced or rate of oxygenate production is cited below as appropriate. Given potential interest in rates in units of mmol product/mmol metal-h, those values are also shown throughout the manuscript.

## 3 Results and discussion

### 3.1 Basic characterization

**Fig. 2:**
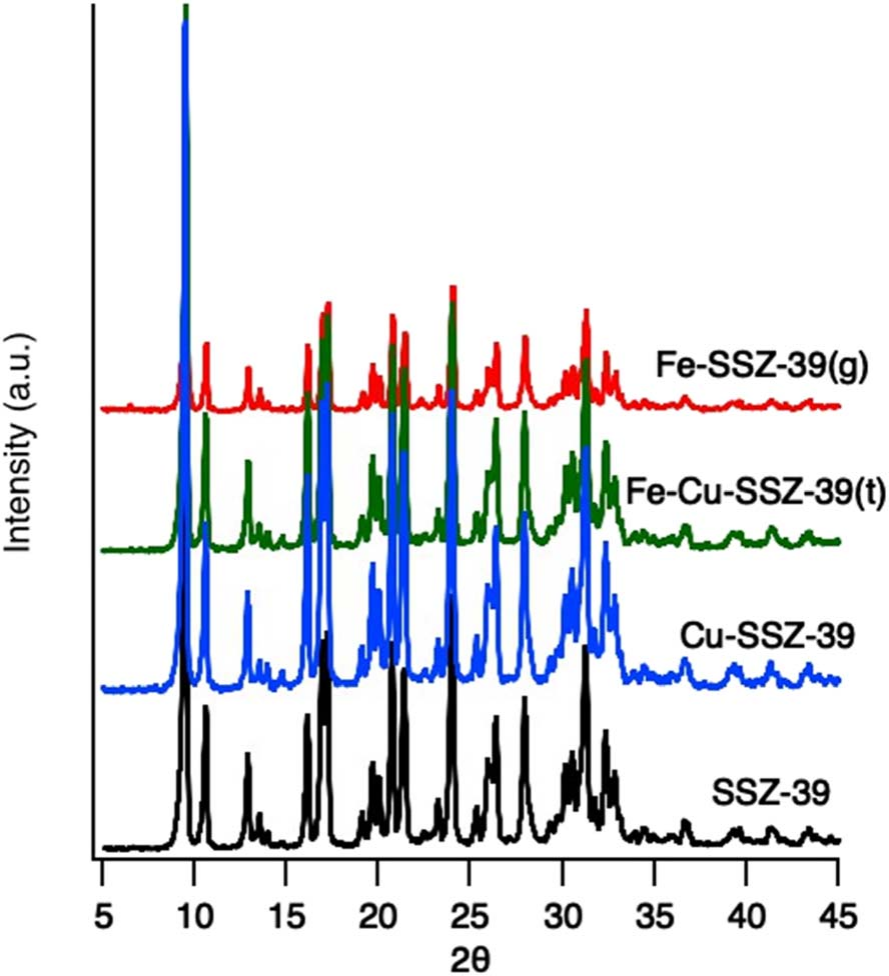
Powder X-ray diffraction pattern of SSZ-39 and exchanged samples.

The phase purity of SSZ-39 was verified using powder X-ray diffraction (PXRD) and compared with the simulated diffraction pattern of the standard monoclinic SSZ-39 framework.^27^ Diffraction measurements performed on metal exchanged SSZ-39 showed no difference in the diffraction patterns compared to the base SSZ-39 (Fig. 2). This is consistent with the zeolite maintaining its crystallinity during metal exchange and indicates the absence of large copper or iron oxide clusters. EDS and XRF show the base SSZ-39 has a Si/Al of 7.5 ± 0.3.

**Fig. 3:**
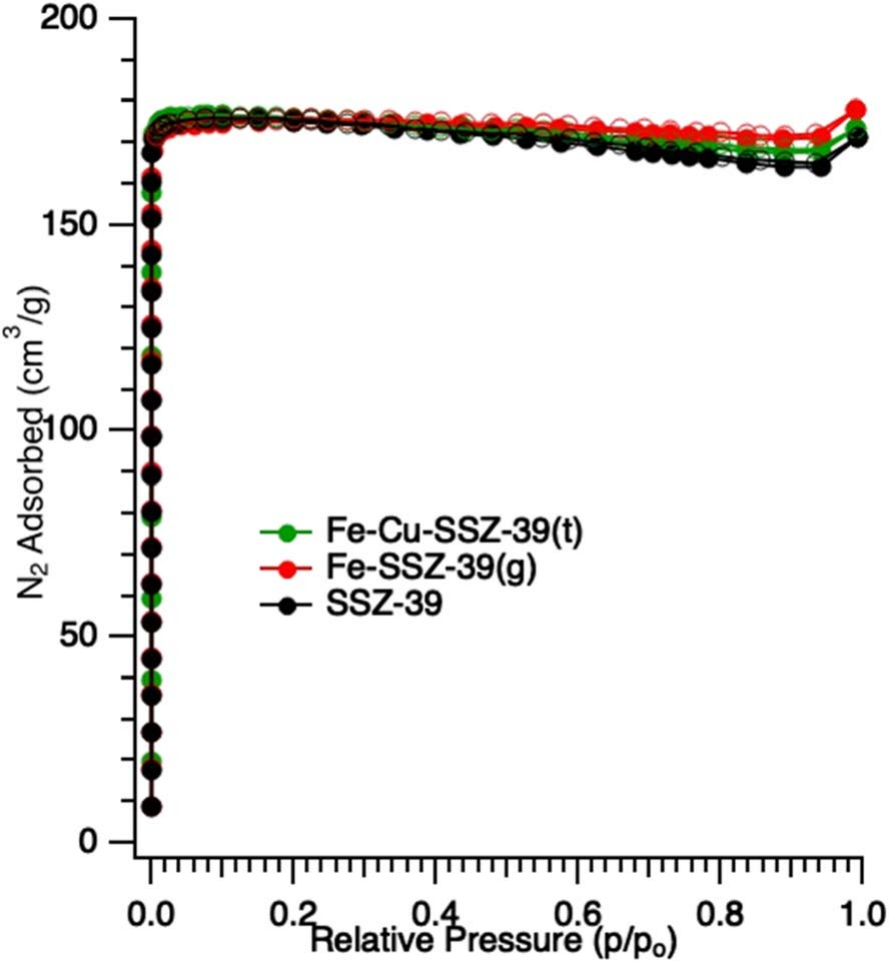
Nitrogen adsorption isotherm of SSZ-39 and exchanged samples.

Nitrogen adsorption measurement of the parent material^36^ displays a type I isotherm (Fig. 3) as expected and possesses a typical micropore volume of 0.28 cm^3^ g^−1^ and BET surface area of 575 m^2^ g^−1^. The measurements on Fe-SSZ-39 and Fe–Cu-SSZ-39 showed no significant differences in the surface area (576 and 573 m^2^ g^−1^) or pore volume (0.27 and 0.28 cm^3^ g^−1^). SEM images (SI Fig. S1) show single homogeneous SSZ-39 crystals of cuboidal morphology with an average crystal size of ∼0.3 µm and shows no appreciable changes in the crystal size or shape for Fe,Cu-SSZ-39. The SEM images of the commercial MFI samples used are included in the SI (Fig. S2). In short, they are irregular shaped submicron particles, of comparable size to the SSZ-39 materials.

### 3.2 Effect of metal loading protocol on material composition

Multiple exchange methods were utilized to load iron and copper into SSZ-39. Table 1 below summarizes the compositions of the materials obtained and the order of metal addition. A few points stand out from Table 1. First, the efforts to exchange iron into Cu-SSZ-39 *via* room temperature exchange (Cu, Fe-SSZ-39(r)) were unsuccessful. The wet impregnation method was problematic as very low amount of salt solution needs to be used and also comes with the assumption of whatever is added goes into the material. The simultaneous exchange of iron and copper into SSZ-39 was successful in that both metals did exchange, however the composition of the product Fe : Cu of approximately 1 : 3 is different from the exchange conditions of 1 : 1, *i.e.*, copper was preferentially exchanged. The last conclusion is that it was possible to make materials with both Fe and Cu in them reproducibly. Given the above, we chose not to catalytically test the samples prepared *via* room temperature exchange and incipient wetness impregnation.

**Table 1:** Summary of the SSZ-39 materials, their composition, and the method of metal loading

Sample	Si/Fe	Fe/Al	Cu/Al	Preparation method
Fe-SSZ-39(g)	187	0.045	0	Gao method^32^
Fe-SSZ-39(g)*	43	0.151	0	Gao method^32^
Fe-SSZ-39(o)	98	0.08	0	One-pot synthesis^31^
Fe,Cu-SSZ-39(t)	116	0.07	0.196	Two step method,^32^ Fe then Cu
Fe,Cu-SSZ-39(t)*	32	0.212	0.031	Two step method,^32^ Fe then Cu
Cu,Fe-SSZ-39(m)	56	0.128	0.085	Modified Gao method^32^, Cu then Fe
Fe/Cu-SSZ-39(mm)	124	0.057	0.189	Simultaneous Cu, Fe metal exchange^26^

### 3.3 Effect of metal loading protocol on the methanol/formic acid production for Fe-SSZ-39 catalysts

**Fig. 4:**
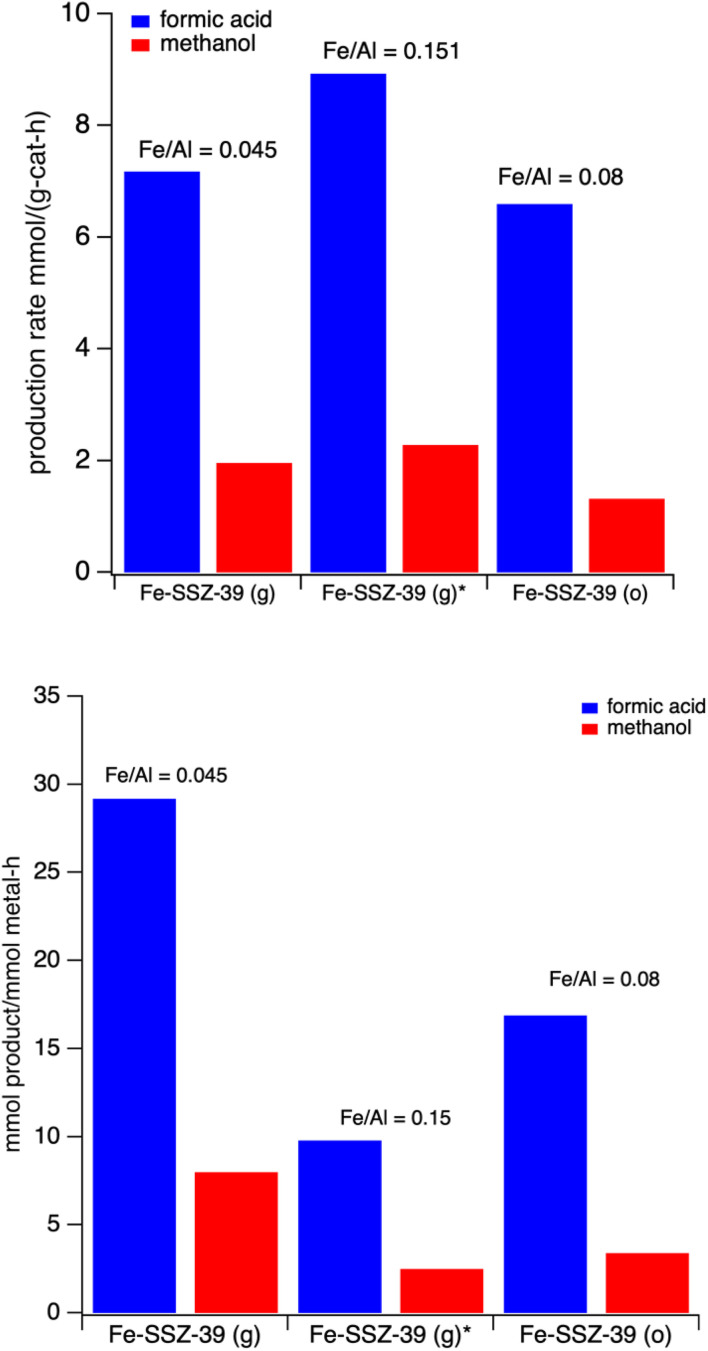
Formic acid (blue) and methanol (red) production (top) per unit mass catalyst per hour and (bottom) per unit mole of metal per for three Fe-SSZ-39 materials. Reaction conditions: 27 mg catalyst, 10 mL 0.5 M H_2_O_2_, methane pressure of 443 psi. Heated at 55 °C for 60 minutes with stirring at ∼1400 rpm.

Three different Fe-SSZ-39 catalysts were initially tested for methanol/formic acid production. First, two samples were prepared using the Gao method but containing different iron contents, and another sample was made *via* the one-pot synthesis method. The formic acid and methanol production for these materials is shown in Fig. 4. Plots showing production normalized by mass catalyst and moles metal are also included in Fig. 4. On a mass catalyst basis, it is observed that the sample with the highest iron content appears to be the most active. Also, on a mass basis the one-pot preparation appears to slightly less active than the low iron content made using the Gao method. However, on a unit mole of metal basis the activity is inversely proportional to iron content, *i.e.*, the lowest iron content is most active on a moles of iron basis. Also, of note is that the formic acid to methanol ratio is very similar for both of the samples made using the Gao method (3.9 and 3.7) despite their very different activity on a unit metal basis.

Finally, it is important to note that the base Cu-SSZ-39 (Cu/Al = 0.214) material did not show any activity for methanol or formic acid production.

### 3.4 Formic acid and methanol production over mixed Fe,Cu SSZ-39

**Fig. 5:**
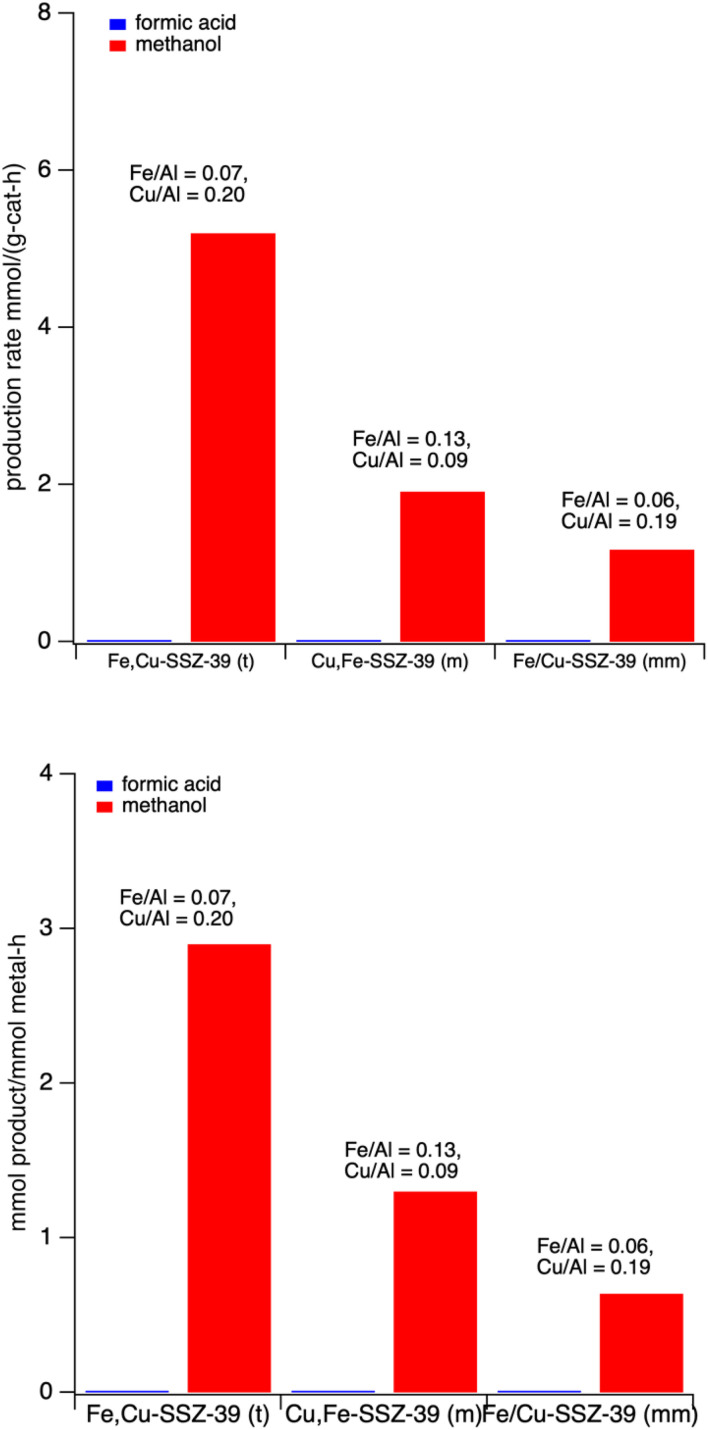
Production rates of methanol and formic acid over three mixed metal SSZ-39 samples (top) on a unit mass catalyst basis and (bottom) on a unit total metal basis. Reaction conditions: 27 mg catalyst, 10 mL 0.5 M H_2_O_2_, methane pressure of 443 psi. Heated at 55 °C for 30 minutes with stirring at ∼1400 rpm.

Fe,Cu-SSZ-39 catalysts prepared from three different methods were then tested for 30 minutes of reaction. Fe,Cu-SSZ-39(t) is prepared by using Fe-SSZ-39 from the Gao method and then copper exchanged; Cu,Fe-SSZ-39(m) is prepared by loading iron *via* the Gao method to Cu-SSZ-39, and Fe/Cu-SSZ-39(mm) is a simultaneous exchange. These results are shown in Fig. 5. A key observation is that all three samples selectively produced methanol, and there is no formic acid observed. Also, within our ability, the materials have a comparable total metal content of M/Al between 0.22 and 0.27 (*vide infra*). The results in Fig. 5 also show that samples where iron is loaded first and then copper (Fe,Cu-SSZ-39(t)) have higher activity then when the metals are loaded in the opposite order. Employing the Gao method to load iron into already formed Cu-SSZ-39, we were able to get Cu, Fe-SSZ-39(m). However, it was much harder to control the iron and copper contents when loading iron into the copper zeolite, *i.e.*, Fe,Cu-SSZ-39(t) gave more reproducible and controllable metal contents than the prep of Cu,Fe-SSZ-39(m). Given that, the Cu,Fe-SSZ-39(m) materials were not pursued further. Another method, simultaneous exchange using two different salts of iron and copper in the same mixture to get Fe/Cu-SSZ-39(mm) was also used. Based on the EDX analysis on these samples, the two-step method (Fe,Cu-SSZ-39(t)) resulted in consistent values of copper and iron content, and thus this method was used to prepare most of the samples for the catalytic testing (Table 1). Given that Fe,Cu-SSZ-39(t) showed both higher methanol production and higher ease in controllable and reproducible metal loading this material was studied in more detail. We will discuss materials with different Cu/Fe ratios and how that impacts production rates and selectivities below (*vide infra*).

### 3.5 Methanol/formic acid production with samples made *via* two-step method

#### 3.5.1 Effect of reaction time

**Fig. 6:**
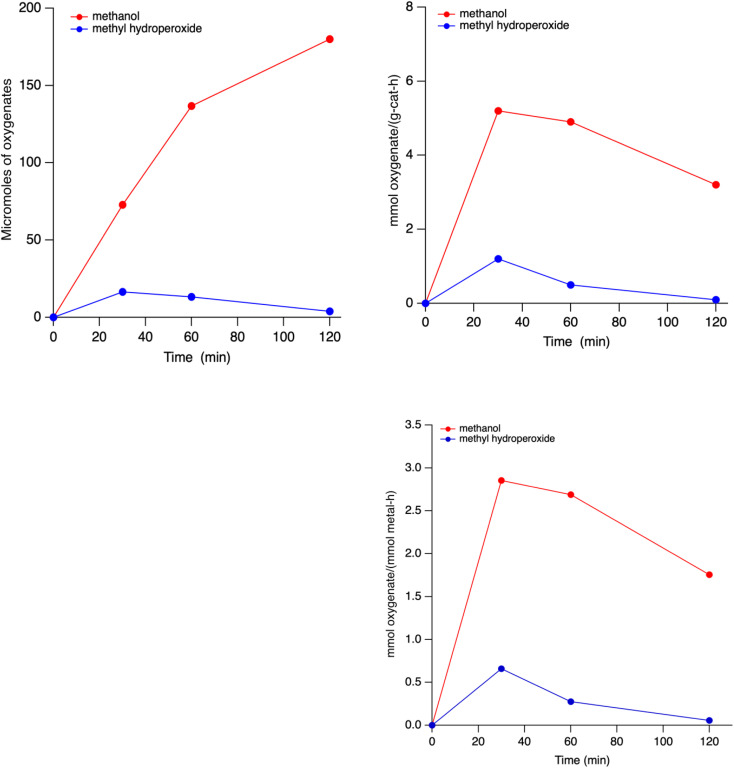
(Clockwise from top left) plot of methanol and methylhydroperoxide present as a function of time of stream. (Top right) production rate of oxygenates as a function of time on stream in units of mass catalyst. (Bottom right) production rate of oxygenates as a function of time on stream in units of moles of metal. Reaction conditions: 27 mg catalyst, 10 mL 0.5 M H_2_O_2_, methane pressure of 443 psi. Heated at 55 °C for time shown with stirring at ∼1400 rpm.

Fe,Cu-SSZ-39(t) catalysts made from the two-step method were reacted with methane in presence of H_2_O_2_ for 30 min to 2 h of reaction time (Fig. 6). The increase in the reaction time from 30 min to 2 h showed a large increase in the methanol production (73 µmmol to 180 µmol) and still no formic acid production (Fig. 5). The complete lack of formic acid production is notable. At lower reaction times, we observed higher methyl hydroperoxide formation that gradually decreased as reaction time was increased suggesting methyl hydroperoxide as an intermediate step towards methanol formation rather than *via* formic acid formation. Reaction times longer than 4 h produced some other compounds due to what we believe is non-selective decomposition of H_2_O_2_. (not shown). The results shown above for the Fe,Cu-SSZ-39(t) samples are samples that had not been calcined prior to catalytic testing. Experiments on samples in the protonic (H^+^) form (after calcination) produced slightly higher amounts of methanol at the same reaction time of 1 h (137 ± 1.29 *vs.* 146 ± 1.57 µmol) suggesting that acid sites help enable the chemistry, albeit only moderately.

#### 3.5.2 Leaching studies

**Fig. 7:**
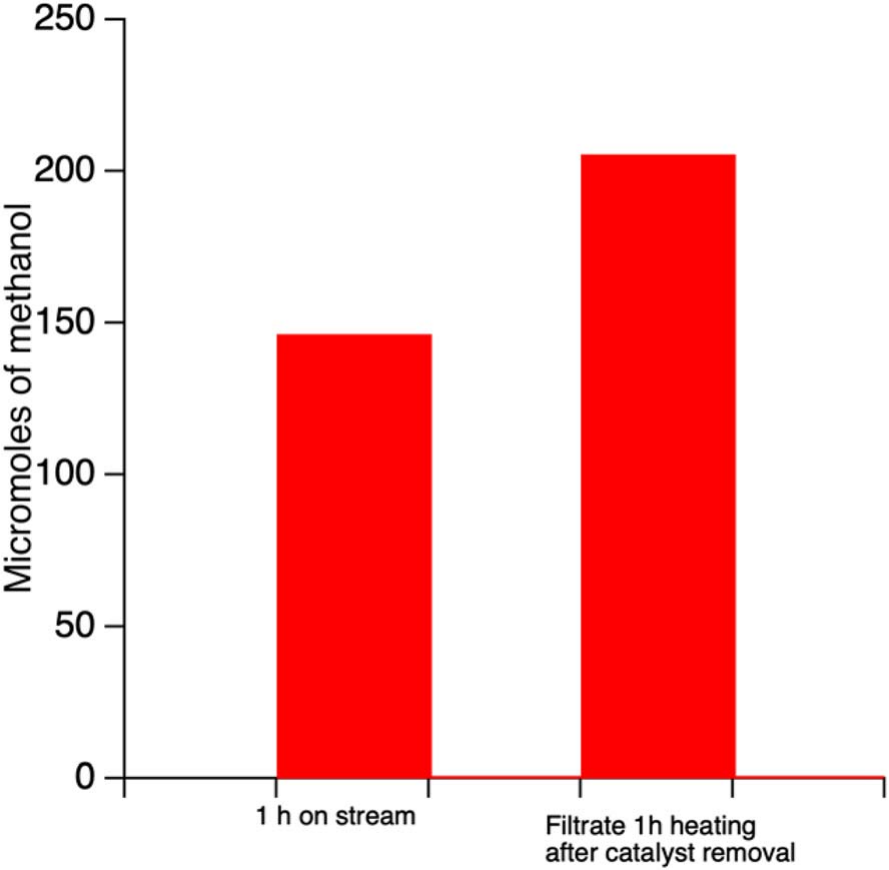
Leaching studies on protonic form of Fe,Cu-SSZ-39(t) catalysts.

In order to investigate the possible leaching of copper and iron, we conducted a so-called “hot-filtrate” experiment where the reaction was carried out for 1 h. After cooling down to room temperature using ice bath, the contents of the reactor were filtered to separate the catalyst. The leftover liquid was then repressurized with methane and reacted for 1 h without adding any catalyst. The liquid products from this reaction when analyzed showed an increase in the amount of methanol present from 146 to 206 µmol (Fig. 7) implying the possible leaching of iron and copper into solution. In contrast, experiments performed where no catalyst was added at the beginning of reaction (*i.e.*, a control) showed no oxygenate production. Controls performed with SSZ-39 (no copper or iron) showed only trace amounts of products. These controls point to the fact that the transition metal is responsible for the observed activity.

#### 3.5.3 Effect of copper and iron content

**Fig. 8:**
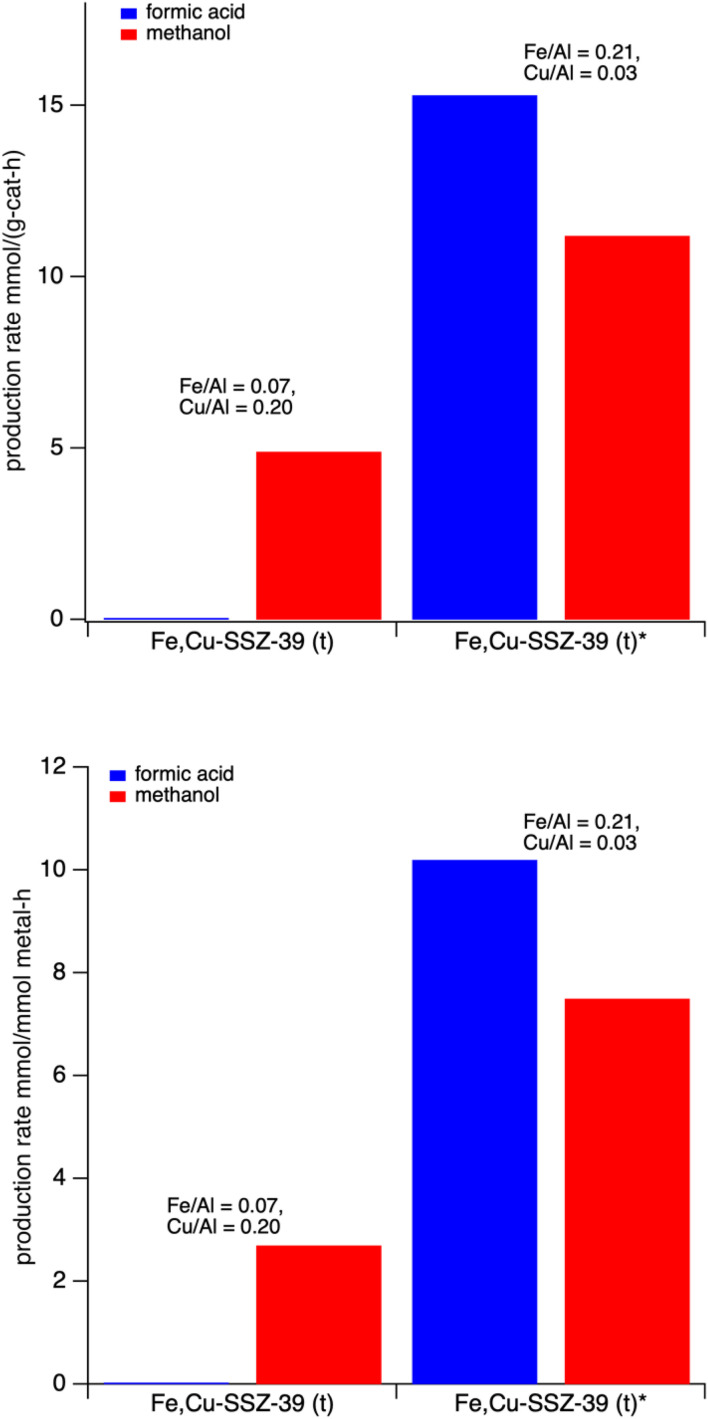
Methanol and formic acid production rates over Fe,Cu-SSZ-39(t) catalysts with varying iron and copper content after 1 h of reaction. (Top) plot on a mass catalyst basis, (bottom) plot ona moles of metal basis. Reaction conditions: 27 mg catalyst, 10 mL 0.5 M H_2_O_2_, methane pressure of 443 psi. Heated at 55 °C for 60 minutes with stirring at ∼1400 rpm.

Fe,Cu-SSZ-39(t) catalysts with different copper and iron content were tested for the liquid phase reaction for 1 hour and their methanol and formic acid production rates are plotted in Fig. 8. Samples with the lowest iron content (Cu/Al = 0.20 and Fe/Al = 0.07) produced only methanol (152 µmol) after 1 hour with no formic acid. By contrast, when the metal loadings were approximately inverted (Fe/Al = 0.21, Cu/Al = 0.03), the catalyst showed the highest formic acid production (429 µmol) as well as methanol production (314 µmol) after 1 hour, resulting in the highest oxygenate production rate observed. This result is striking for two reasons. First, at a comparable metal content (M/Al between 0.24–0.27) the product selectivity not only varies dramatically but the product yield is markedly improved. Second, in comparing the Fe,Cu-SSZ-39(t)* to the sample the pure Fe-SSZ-39(g)* sample in Fig. 3, the mixed metal sample has more iron by 40% than the pure iron sample yet is still more active on a unit total metal basis. This very interesting results shows that at comparable total metal (Fe + Cu) content the relative production rates of methanol and formic acid can be affected.

### 3.6 Comparison to Cu–Fe-ZSM-5(topology/porosity effects)

**Fig. 9:**
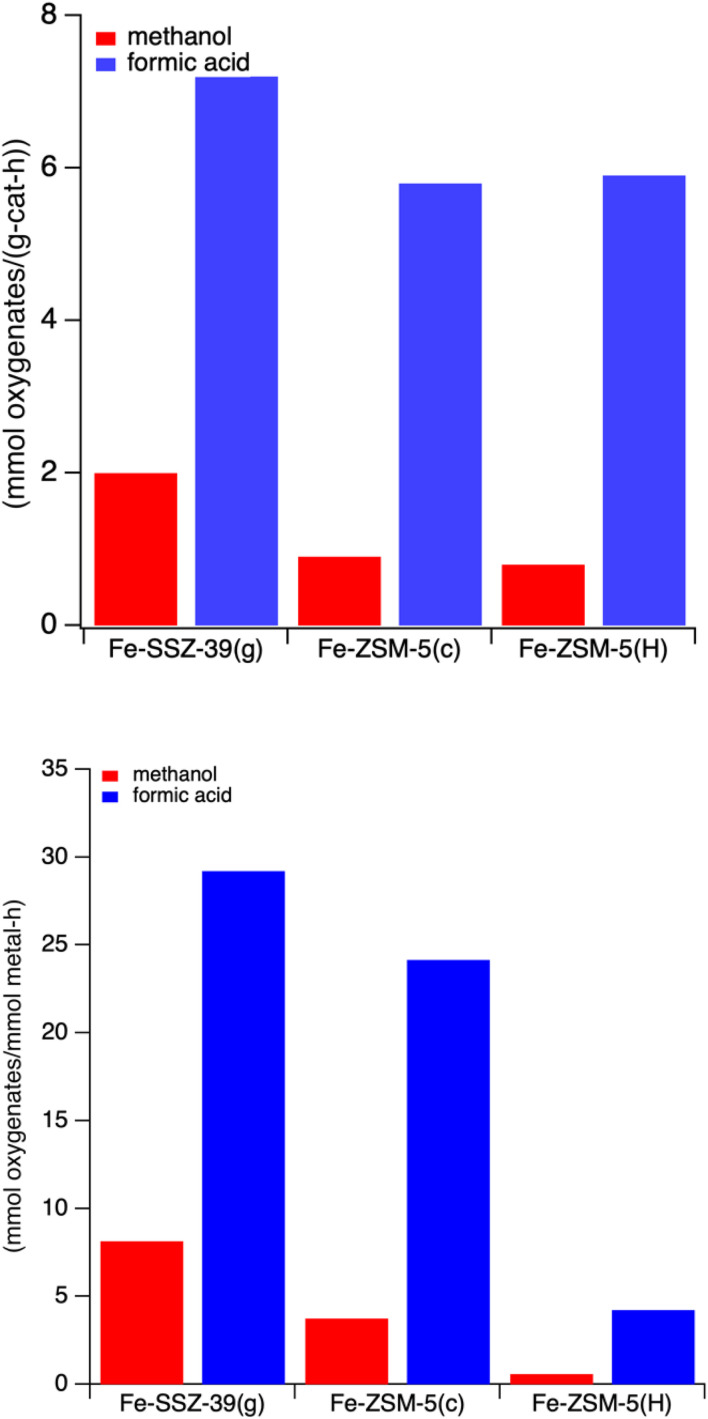
(Top) Methanol and formic acid production rates over Fe-SSZ-39 *vs.* Fe-ZSM-5 catalysts after one hour of reaction time on a unit mass catalyst basis. (Bottom) methanol and formic acid production rates over Fe-SSZ-39 *vs.* Fe-ZSM-5 catalysts after one hour of reaction time on a unit mole total metal basis. Reaction conditions: 27 mg catalyst, 10 mL 0.5 M H_2_O_2_, methane pressure of 443 psi. Heated at 55 °C for 60 minutes with stirring at ∼1400 rpm.

To the best of our knowledge this is the first report in the open literature exploring liquid-phase methane oxidation over Fe–Cu small-pored zeolites. Given that, we wished to compare these findings with other zeolite-based catalysts, specifically Fe- and Fe,Cu-ZSM-5. Fe-ZSM-5 and Fe,Cu-ZSM-5 catalysts have been studied for liquid phase oxidation with methane by Hammond and coworkers and observed methyl hydroperoxide as an intermediate which decomposes to form methanol and formic acid.^20^ In addition, prior work from our lab by Shahami and Shantz on the liquid-phase oxidation of methane over Fe-ZSM-5 catalysts showed production of different oxygenates with formic acid as the main component.^35^ Here, Fe-SSZ-39(g), Fe-ZSM-5(c) and Fe-ZSM-5(H) from Hammond's work are compared in Fig. 9. In all three sets of materials, iron-exchanged zeolites favor formic acid over methanol. In addition, Fe-SSZ-39 show better reactivity than Fe-ZSM-5 in terms of both methanol (∼55 µmol) and formic acid (∼200 µmol). Both Fe-ZSM-5 samples show similar methanol (∼25 µmol) and formic acid (∼165 µmol) production.

**Fig. 10:**
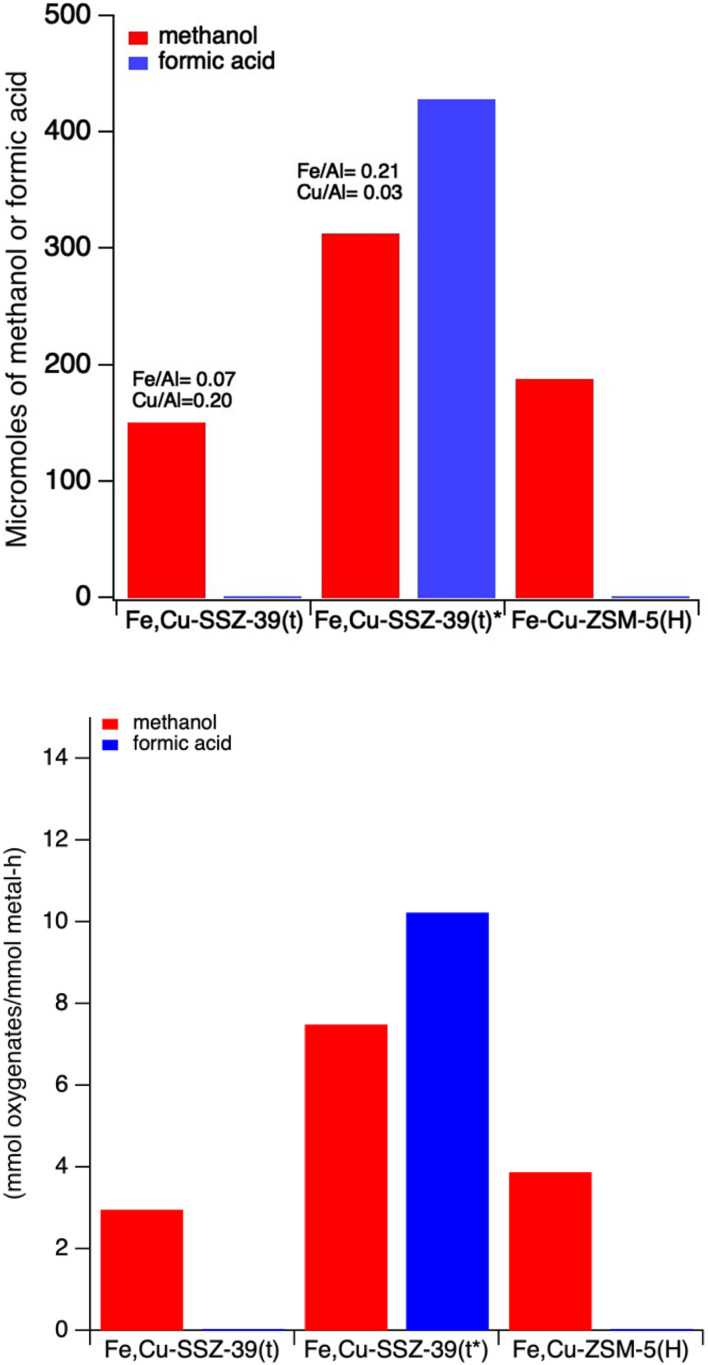
(Top) methanol and formic acid production over Fe,Cu-SSZ-39 *vs.* Fe–Cu-ZSM-5 catalysts after one hour of reaction time on a unit mass catalyst basis. (Bottom) methanol and formic acid production over Fe,Cu-SSZ-39 *vs.* Fe–Cu-ZSM-5 catalysts after one hour of reaction time on a unit total metal basis. Reaction conditions: 27 mg catalyst, 10 mL 0.5 M H_2_O_2_, methane pressure of 443 psi. Heated at 55 °C for 60 minutes with stirring at ∼1400 rpm.

In comparing the mixed metal ZSM-5 and SSZ-39 catalysts in Fig. 9, we observed that Fe–Cu-ZSM-5(H) from Hammond's work^26^ produced slightly more methanol than Fe,Cu-SSZ-39(t) without any formic acid at low iron content. However, when we increase the iron content to Fe/Al = 0.212 (Fe,Cu-SSZ-39(t)*), we observed higher methanol production as well as formic acid (Fig. 10).

## 4 Conclusions

Iron ions in zeolite SSZ-39 favor formic acid formation while iron with copper shifts the selectivity towards methanol with no observable formic acid formation. The product distribution of oxygenates from these experiments included methanol, formic acid, methyl hydroperoxide. An interesting finding here is that by adding copper to Fe-SSZ-39 with low iron contents it is possible to eliminate the production of formic acid and selectively make methanol. Cu–Fe-SSZ-39(t) (Cu/Al = 0.196 and Fe/Al = 0.07) in H^+^ form showed the highest methanol production and Fe,Cu-SSZ-39(t) (Fe/Al = 0.212, Cu/Al = 0.031) showed highest formic acid production as well as total amount of oxygenates. This work has shown that SSZ-39 samples under the same reaction conditions are capable of producing more oxygenates than ZSM-5.

## Conflicts of interest

There are no conflicts to declare.

## Supplementary Material

RA-015-D5RA04892C-s001

## Data Availability

The data supporting this article can be found in the supplementary information (SI). Supplementary information: SEM images of copper and iron containing zeolites. See DOI: https://doi.org/10.1039/d5ra04892c.
