# Supplementary material for: Partial oxidation of methane over SSZ-39 zeolites containing iron, copper, and iron–copper mixtures with hydrogen peroxide: selective control of oxygenate formation
Source: RSC Adv. 2025 Nov 25;15(54):46345–55. doi: 10.1039/d5ra04892c (PMC12645279; doi:10.1039/d5ra04892c)
Supplement: RA-015-D5RA04892C-s001 [file RA-015-D5RA04892C-s001.pdf]

Supplementary Information (SI) for RSC Advances.
This journal is © The Royal Society of Chemistry 2025

Partial Oxidation of Methane over SSZ-39 Zeolites Containing Iron, Copper, and Iron-Copper Mixtures with Hydrogen Peroxide: Selective Control of Oxygenate Formation

Jeewan Pokhrel and Daniel F. Shantz*

Department of Chemical and Biomolecular Engineering,
Tulane University
6823 St. Charles Avenue, New Orleans, LA 70118, United States

Supporting Information

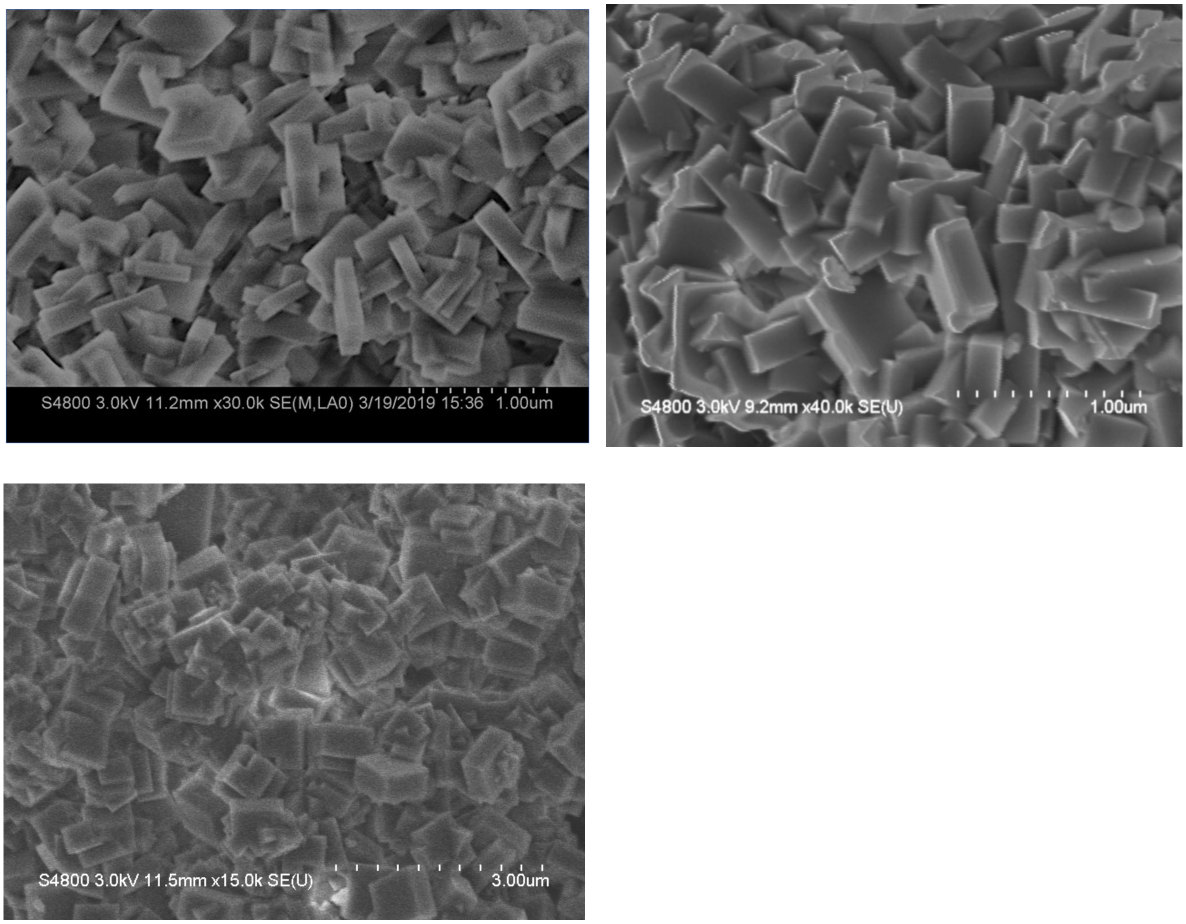

Figure S1 SEM images of SSZ-39, Cu-SSZ-39, Fe-Cu-SSZ-39

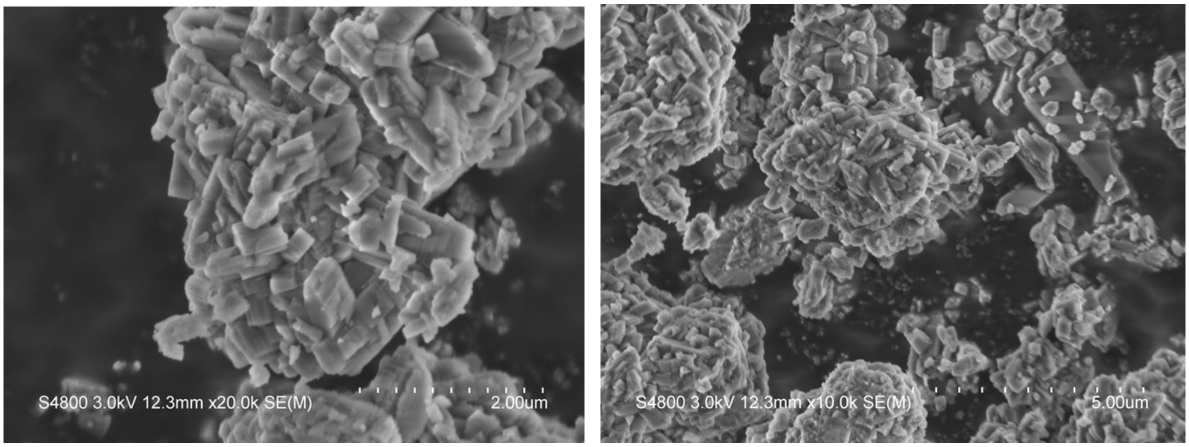

Figure S2 SEM images of commercial ZSM-5
